# Machine learning algorithms for predicting days of high incidence for out-of-hospital cardiac arrest

**DOI:** 10.1038/s41598-023-36270-6

**Published:** 2023-06-19

**Authors:** Kaoru Shimada-Sammori, Tadanaga Shimada, Rie E. Miura, Rui Kawaguchi, Yasuo Yamao, Taku Oshima, Takehiko Oami, Keisuke Tomita, Koichiro Shinozaki, Taka-aki Nakada

**Affiliations:** 1 Department of Emergency and Critical Care Medicine, Chiba University Graduate School of Medicine, 1-8-1 Inohana, Chuo, Chiba, 260-8677 Japan; 2 Smart119 Inc, 2-5-1, Chuo, Chiba, Japan; 3 Department of Emergency Medicine, Zucker School of Medicine, New York, USA

**Keywords:** Cardiovascular biology, Risk factors, Machine learning, Predictive medicine

## Abstract

Predicting out-of-hospital cardiac arrest (OHCA) events might improve outcomes of OHCA patients. We hypothesized that machine learning algorithms using meteorological information would predict OHCA incidences. We used the Japanese population-based repository database of OHCA and weather information. The Tokyo data (2005–2012) was used as the training cohort and datasets of the top six populated prefectures (2013–2015) as the test. Eight various algorithms were evaluated to predict the high-incidence OHCA days, defined as the daily events exceeding 75% tile of our dataset, using meteorological and chronological values: temperature, humidity, air pressure, months, days, national holidays, the day before the holidays, the day after the holidays, and New Year’s holidays. Additionally, we evaluated the contribution of each feature by Shapley Additive exPlanations (SHAP) values. The training cohort included 96,597 OHCA patients. The eXtreme Gradient Boosting (XGBoost) had the highest area under the receiver operating curve (AUROC) of 0.906 (95% confidence interval; 0.868–0.944). In the test cohorts, the XGBoost algorithms also had high AUROC (0.862–0.923). The SHAP values indicated that the “mean temperature on the previous day” impacted the most on the model. Algorithms using machine learning with meteorological and chronological information could predict OHCA events accurately.

## Introduction

Out-of-hospital cardiac arrest (OHCA) is a public health issue worldwide, and survival after OHCA remains unsatisfactory^1^. Prevention or early recognition using scoring systems has been emphasized in in-hospital cardiac arrest (IHCA) and pediatric cardiac arrest guidelines^2, 3^. Accordingly, prevention and early recognition of OHCA in adult patients are important; prediction algorithms for OHCA events may improve OHCA outcomes through prevention or early recognition.

Baseline characteristics, which are known as risk factors for OHCA, include older age, male sex, past medical history or family history of coronary heart disease, sudden cardiac arrest, high blood pressure, and dyslipidemia^4–6^. In addition, external factors such as weather conditions and human chronological behaviour patterns are also known risk factors for OHCA incidence. Specifically, OHCA incidence tends to increase in winter, on days with cold ambient temperatures and large diurnal temperature ranges^7^, or in the early morning, on weekends^8^. Although these external risk factors of OHCA incidence have been elucidated, few studies have used prediction algorithms for OHCA using both meteorological and chronological data.

In recent years, machine learning techniques have rapidly developed as diagnostic and prognostic tools. A machine learning algorithm can assist in the quick and automatic learning of data patterns, even from complex data. Machine learning approaches have shed new light on resuscitation science.

Thus, we hypothesized that machine learning algorithms using meteorological and chronological information can be used to accurately predict high OHCA incidence and help clinicians identify “high-risk” days for OHCA incidence. We used a large sample size of the OHCA cohort (> 136,000 patients) from the Japanese population-based repository database and tested the accuracy of our algorithms. Because machine learning approaches can identify key factors, we performed a deeper analysis of the association of these factors.

## Methods

### Study setting and patients

**Figure 1:**
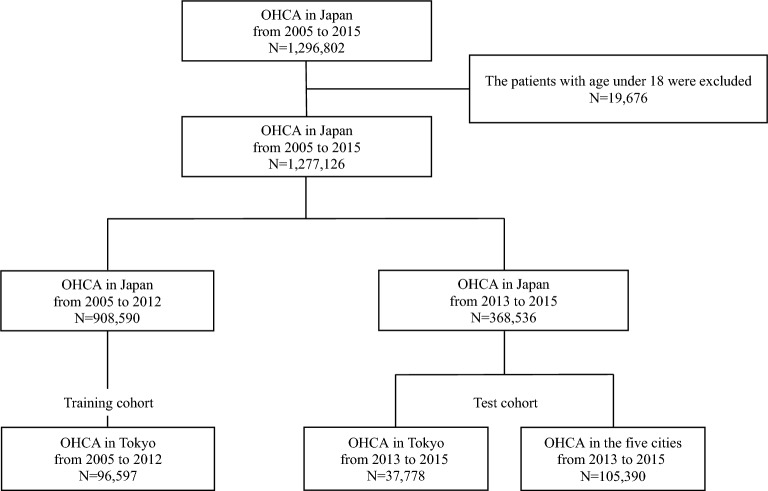
Flowchart of study enrollment.

The nationwide, population-based OHCA registry by the Fire and Disaster Management Agency of the Ministry of Internal Affairs and Communications, in the form of the internationally standardized Utstein style, Japan^9^, between January 2005 and December 2015, was collated. Despite their etiologies (e.g., presumed cardiac diseases, respiratory diseases and trauma), all OHCA events were included. In this database, patients who were not transported to the hospital due to distinct postmortem changes were excluded from registration. The definition of the distinct postmortem change for patients with non-transportation after a cardiac arrest based on the Japanese law satisfies all of the followings: (1) pupil dilation, (2) loss of reflection to the light of pupils, (3) loss of body temperature, (4) appearance of rigour mortis or postmortem lividity. Of the 1,296,802 patients, 1,277,126 patients aged 18 years and older were included in the study. Since we assume that urban areas of mainland Japan have similar baseline characteristics of population composition and weather, we chose datasets from the top six populated prefectures, including Tokyo, Kanagawa, Aichi, Osaka, Saitama, and Chiba. (Supplementary Fig. 1). In the training cohort, we included 96,597 patients in Tokyo from January 2005 to December 2012. We included 143,168 patients in the top six populated prefectures (37,778 in Tokyo and 105,390 in the other five cities) from January 2013 to December 2015 as test cohorts (Fig. 1).

The Chiba University Hospital Certified Clinical Research Review Board approved this study (No. 4042) and waived the need for written informed consent in accordance with the Ethical Guidelines for Medical and Health Research Involving Human Subjects in Japan.

### Definition and data collection

We had to determine the accurate time of OHCA to clarify OHCA incidence. However, the exact time of the OHCA incidence was uncertain in not witnessed patients, and the OHCA incidence may be the OHCA recognition. In this study, the patients with distinct postmortem changes were excluded. As the changes would be found a half day after CA, almost all OHCA patients in this study must be recognized at least half a day after CA. Therefore, we focused on day of the OHCA incidence and identify “high risk” days for OHCA incidence.

The OHCA incidence per one million people was calculated for each prefectural population using annual population data from the e-Stat Portal Site of *Official Statistics of Japan* from 2004 to 2016 (https://www.e-survey.go.jp/en)*.* We defined the OHCA high-incidence days as those exceeding the number of OHCA events greater than 75% tile of the Tokyo data between 2005 and 2015. In addition, we examined the number exceeding 95% tile of the OHCA events in the Tokyo data.

For the prediction of high-incidence days of OHCA, explainable features such as the percentage of the elderly population (per prefecture), date of the emergency call, meteorological conditions and chronological features were used to train the machine learning models. The meteorological data included temperature, humidity, and air pressure. For the chronological features, we included months, days, national holidays, the day before the holidays, the day after the holidays, and New Year's holidays (between 29th December and 3rd January) in the final model, according to previous studies^10^.

Weather data were obtained from the Japan Meteorological Agency and are available to the public (http://www.data.jma.go.jp/gmd/risk/obsdl/index.php). The weather data at the meteorological observatory located in the city with the largest population in each prefecture was chosen as the representative weather data for each prefecture.

### Feature selection

The meteorological predictors considered in this study were the daily average, minimum, maximum, and diurnal changes in ambient temperature, relative humidity, and air pressure. Our strategy for feature selection is to identify the best combination of meteorological features that predict daily OHCA incidence and then to use those features to predict high-incident OHCA days in the final classification model. As weather conditions are expected to affect human health with a time lag, we also estimated the best time lags for each meteorological feature.

For the selection of the meteorological features, we took multiple steps to identify the features that correlate with OHCA occurrence by building a Poisson univariate model by combining each meteorological feature with an estimated time lag and the number of OHCA occurrences per day (Supplementary Methods for details). Finally, the following two meteorological variables were selected as the best predictive features: the average and diurnal temperature of the previous day. We then used these features with all the chronological features mentioned above, as well as the percentage of the elderly population, as input features of the machine learning models.

### Statistical analysis

The primary outcome variable was the number of high-incidence OHCA days. Various machine learning algorithms were used to conduct the primary analysis of OHCA prediction in the Tokyo training cohort. The eight algorithms included XGBoost, RF, LDA, LR, SVM, NB, MLP, and KNN as a representative of a gradient boosting algorithm, tree algorithm, dimension reduction technique, linear algorithm, Bayes theorem-based algorithm, neural network algorithms and k-nearest neighbours algorithm, respectively. These algorithms were selected based on their ability to identify different combinations of features, including linear and nonlinear combinations. For example, XGBoost and MLP can identify linear and nonlinear combinations of features, while LDA and LR can identify linear combinations of features. RF, NB, SVM and KNN can identify nonlinear combinations. By utilizing these algorithms, we aimed to carefully select the most effective model for predicting OHCA using a combination of meteorological and demographic information.

We built these eight classification models with fine-tuned parameters using the training data and evaluated the performance of the eight models based on AUROC values, which were estimated from sixfold cross-validation. In cross-validation, the folds are split into chronological sets as a standard procedure when handling time-series data. Each fold for the training and test data has a fixed time interval of five years and one year, respectively. Of the eight machine-learning algorithms in the training cohort, the best machine-learning algorithm, XGBoost, was used in the test cohort. Additionally, we investigated whether the XGBoost model could predict the OHCA events even if the value of 95% tile was used as the threshold. A sub-group analysis was conducted in patients with OHCA presumably caused by cardiac diseases in the most predictive model, the XGBoost model.

The performance of the models was measured in terms of the AUROC as a superior metric as well as the accuracy, sensitivity, specificity, and F1 score. We used the SHAP algorithm of the XGBoost model to interpret the contribution of each feature to the predictive model. In the algorithm, the SHAP value was computed by the difference in the model output resulting from including a feature in the algorithm, providing information about the impact of each feature on the output. In the SHAP summary plots, every violin plot is composed of all the data points from each feature, with a higher value being redder and a lower value being bluer. The violin plots were aligned with the SHAP values along the x-axis. Thus, a redder/bluer violin plot on the right side (i.e., a higher positive SHAP value) suggests that the higher/lower the value of the feature, the more the model predicts a positive/negative impact.

Since the SHAP analysis revealed important factors, including temperature, percentage of the elderly population, onset day, and month, we investigated the incidence of daily OHCA between elderly and non-elderly patients as a secondary analysis using Tokyo data from 2005 to 2015. The elderly population was defined as people aged 65 and over in this study based on the popular age for retirement to compare the influence of chronological behaviour differences.

In this study, we used the nationwide, population-based OHCA registry, which collects OHCA data from the entire population under investigation. We used all the data that is available, therefore sample size calculation was not applicable. Data are expressed as medians (interquartile ranges) for continuous values and absolute numbers and percentages for categorical values. We employed the Mann–Whitney U test for numerical variables, and the Chi-square test for categorical variables. Statistical significance was set at *p* < 0.05.

Analyses were performed using Python 3.7.6 packages (Scikit-learn 0.23.2, XGBoost 1.1.1, Pandas 1.1.5, and NumPy 1.19.2) to construct machine learning models. Python packages, including Scikit-learn, XGBoost, Pandas, Numpy, Matplotlib, and the SHAP package are all open-source packages. Permission to use these packages is granted free of charge to any person (Python License: https://docs.python.org/3/license.html, SHAP: https://github.com/slundberg/shap/blob/master/LICENSE). All the figures in this study were created using the visualization package in Python Matplotlib (3.3.4)^11, 12^.

## Results

### Baseline characteristics and meteorological features

From January 2005 to December 2015, the number of patients of OHCA in Japan was 1,296,802, of which 1,277,126 were above 18 years old. The etiologies were presumed cardiac diseases (730,585, 57%), respiratory diseases (83,299, 6%), exogeneous (184,521, 34%), traffic accident and so on. The training cohort included 96,597 OHCA patients (Tokyo, 2005–2012) (Table 1); the median daily incidence of OHCA was 2.5 cases per one million. In the test cohorts (six prefectures, 2013–2015), the median daily incidence of OHCA ranged from 2.3 to 2.5 cases per one million. The percentage of the elderly (65 years or older) population in the six cities ranged from 19.8 to 24.5%, and the percentages of elderly OHCA patients ranged from 71.9 to 80.5% in the six cities. There were no statistically significant differences in the annual average temperature, humidity, or air pressure between the six prefectures.

**Table 1:** Characteristics of patients and weather data in training and test cohort.

Training/test data	Tokyo	Tokyo	Kanagawa	Osaka	Aichi	Saitama	Chiba
	Training data	Test data	Test data	Test data	Test data	Test data	Test data
Collecting year	2005–2012	2013–2015	2013–2015	2013–2015	2013–2015	2013–2015	2013–2015
Collecting days	2922	1095	1095	1095	1095	1095	1095
Patients total n	96,597	37,778	25,842	22,655	20,425	19,832	16,636
Elderly-n(%)	69,457 (71.9)	28,606 (75.7)	20,300 (78.6)	17,556 (77.5)	16,452 (80.5)	15,737 (79.4)	13,078 (78.6)
Age-yr	76 (62–85)	78 (65–86)	78 (66–86)	77 (66–85)	79 (68–86)	78 (67–85)	78 (66–85)
Male sex-n(%)	56,173 (58.6)	21,860 (58.4)	14,718 (57.3)	12,807 (56.8)	11,362 (56.0)	11,411 (57.8)	9,615 (58.1)
OHCA high-incidence days	852 (29.2%)	285 (26.0%)	310 (28.3%)	191 (17.4%)	282 (25.8%)	280 (25.6%)	258 (23.6%)
Daily OHCA incidence	2.5 (2.1–3.11)	2.5 (2.0–3.0)	2.4 (2.0–3.1)	2.3 (1.8–2.7)	2.4 (1.9–3.1)	2.3 (1.8–3.0)	2.4 (1.8–2.9)
Population-n*	12,514,108	13,242,645	9,105,407	8,871,917	7,481,435	7,293,831	6,249,898
Elderly*-%	19.8	21.7	22.5	24.5	22.5	23.1	23.9
Temperature-℃							
Mean	17.2	17.8	17.4	17.8	17.2	16.6	17.7
Minimum	13.9	14.2	14.2	14.2	13.5	12.2	14.3
Max	20.0	20.8	20.5	21.4	21.2	20.8	20.4
Diurnal	5.9	6.5	6.2	6.3	7.4	8.7	6.0
Humidity-%							
Mean	61.3	63.8	68.7	61.8	62.8	61.5	66.2
Minimum	47.0	47.0	51.0	46.0	44.0	40.0	50.0
Max	77.0	83.0	86.0	79.0	82.0	84.0	83.0
Diurnal	26.0	31.0	29.0	31.0	36.0	38.0	27.0
Air pressure-hPa							
Mean	1010	1010	1008	1005	1007	1009	1012
Minimum	1007	1007	1006	1003	1005	1007	1010
Max	1012	1013	1011	1008	1010	1013	1015
Diurnal	4.7	4.9	4.5	3.9	4.0	4.5	4.2

Elderly patients were defined as those aged > 65 years, OHCA, and out-of-hospital cardiac arrest. *Average number of people per year.

Data are presented as a median and interquartile range for continuous variables. The weather data are presented as a median.

The OHCA high-incidence days, which was defined as the number exceeding 75% tile of daily OHCA incidence in the Tokyo data, was 3.1 per one million population. The other five prefectures had a similar 75% tile of OHCA incidence, which ranged from 2.7 to 3.1 per one million population. High-incidence days had decreased temperature, humidity, and increased air pressure compared to low-incident days; “Month” and “Day” were statistically different in a training cohort (Table 2). In addition, the number exceeding 95% tile was 4.0, which was similar to the 75% tile figure.

**Table 2:** Comparison of meteorological and time data between OHCA high-incidence and not high-incidence days in training data of Tokyo.

	High-incidence days	Not high-incidence days	*P*-value
Sample size-days	852 (29.2%)	2070 (70.8%)	–
Temperature-℃			
Mean	7.6 (5.7–11.2)	19.8 (13.6–24.8)	< 0.001
Minimum	4.6 (2.6–8.0)	17.1 (10.4–22.3)	< 0.001
Max	11.1 (8.9–14.8)	22.9 (17.1–28.0)	< 0.001
Diurnal temperature	6.4 (4.8–7.9)	6.0 (4.5–7.4)	< 0.001
Humidity-%			
Mean	48.1 (37.5–61.7)	65.2 (54.4–72.8)	< 0.001
Minimum	32.0 (23.0–46.0)	49.5 (37.0–59.0)	< 0.001
Max	67.0 (54.0–82.0)	80.0 (72.0–86.0)	< 0.001
Diurnal	29.0 (21.0–38.0)	28.0 (21.0–36.0)	< 0.001
Air pressure-hPa			
Mean	1012 (1006–1016)	1009 (1004–1013)	< 0.001
Minimum	1008 (1003–1013)	1006 (1001–1011)	< 0.001
Max	1015 (1010–1020)	1012 (1007–1016)	< 0.001
Diurnal	6.1 (4.2–9.2)	4.6 (3.3–7.2)	< 0.001
Month	3 (2–11)	7 (5–9)	< 0.001
Day-n(%)			
Holidays	200 (26.8)	388 (17.9)	< 0.001
The day before holidays	113 (15.0)	385 (17.7)	0.100
The day after holidays	150 (20.1)	303 (14.0)	< 0.001
New year holidays	45 (6.0%)	2 (0.1%)	< 0.001
Monday	147 (19.7%)	270 (12.4%)	< 0.001
Tuesday	95 (12.7%)	321 (14.8%)	0.185
Wednesday	104 (13.9%)	312 (14.4%)	0.815
Thursday	92 (12.3%)	324 (14.9%)	0.091
Friday	88 (11.8%)	329 (15.2%)	0.028
Saturday	95 (12.7%)	322 (14.8%)	0.175
Sunday	125 (16.8%)	292 (13.5%)	0.031

High-incidence days were defined as the number of OHCA events greater than 75% of the Tokyo training data.

Data are presented as a median and interquartile range for continuous variables.

*P-values* were calculated using Pearson’s chi-square test or the Mann–Whitney U test.

### Prediction of OHCA high-incidence days

In the primary analysis of OHCA prediction in the Tokyo training cohort using eXtreme Gradient Boosting (XGBoost), random forest (RF), linear discriminant analysis (LDA), logistic regression (LR), multilayer perceptron (MLP), Naïve Bayes (NB), support vector machine with radial basis function kernel (SVM), and k-Nearest Neighbors (kNN), all classification models achieved the mean area under the receiver operating characteristic curve (AUROC) over 0.89 (Table 3). XGBoost had the highest predictive value (AUROC 0.906 [confidence interval; CI 0.868–0.944]), which was chosen for further validation. The developed prediction algorithm demonstrated a high predictive value by repeating the analysis in the test cohorts. The AUROC were similarly high and 0.923, 0.882, 0.888, 0.889, 0.879, and 0.862, respectively in the top six populated prefectures (Table 4). Furthermore, the XGBoost model developed a highly accurate prediction model even if the value of 95% tile was used as the threshold: the AUROC of the training cohort was 0.941, and that of the test cohort was 0.958. In the presumed cardiac diseases of OHCA, the prediction algorithm with the XGBoost model had a high predictive value (the AUROC 0.891, and 0.790–0.859 respectively in training and test cohort) (Supplementary Table 1).

**Table 3:** Predicting the value of high-incidence days using machine learning in the training data of Tokyo.

	AUROC (95% CI)	Accuracy	Sensitivity	Specificity	F1-score
XGBoost	0.906 (0.868–0.944)	0.835 (0.806–0.864)	0.848 (0.778–0.918)	0.833 (0.796–0.871)	0.734 (0.682–0.786)
RF	0.904 (0.866–0.943)	0.838 (0.814–0.862)	0.838 (0.759–0.918)	0.841 (0.811–0.870)	0.735 (0.692–0.778)
LDA	0.903 (0.858–0.948)	0.842 (0.794–0.890)	0.826 (0.790–0.862)	0.849 (0.788–0.911)	0.740 (0.681–0.799)
LR	0.904 (0.856–0.952)	0.832 (0.788–0.875)	0.858 (0.778–0.937)	0.825 (0.762–0.888)	0.733 (0.675–0.791)
SVM	0.905 (0.858–0.952)	0.839 (0.797–0.880)	0.846 (0.793–0.899)	0.837 (0.786–0.888)	0.740 (0.691–0.789)
NB	0.901 (0.856–0.947)	0.843 (0.800–0.886)	0.831 (0.770–0.892)	0.848 (0.791–0.905)	0.742 (0.701–0.783)
MLP	0.904 (0.859–0.950)	0.834 (0.798–0.869)	0.849 (0.794–0.903)	0.829 (0.793–0.864)	0.734 (0.691–0.777)
KNN	0.899 (0.855–0.944)	0.839 (0.809–0.869)	0.832 (0.771–0.893)	0.840 (0.801–0.878)	0.736 (0.699–0.773)

Tokyo data (2005–2012) was analyzed.

*AUROC* area under the receiver operating characteristic curve, *CI* confidence interval, *XGBoost* extreme gradient boosting, *RF* random forest, *LDA* linear discriminant analysis, *LR* logistic regression, *SVM* support vector machine with radial basis function kernel, *NB* naïve Bayes, *MLP* multilayer perceptron, *kNN* k-nearest neighbors.

**Table 4:** Predicting the value of high-incidence days using XGBoost in the test data of the top-six population prefectures.

	AUROC (95%CI)	Accuracy	Sensitivity	Specificity	F1-score
Tokyo	0.923 (0.905–0.938)	0858 (0.835–0.879)	0.841 (0.793–0.884)	0.864 (0.838–0.888)	0.732 (0.689–0.770)
Kanagawa	0.882 (0.860–0.904)	0.804 (0.778–0.826)	0.800 (0.746–0.844)	0.805 (0.775–0.832)	0.668 (0.625–0.707)
Osaka	0.888 (0.863–0.911)	0.748 (0.721–0.774)	0.890 (0.843–0.936)	0.723 (0.694–0.751)	0.514 (0.463–0.562)
Aichi	0.889 (0.863–0.912)	0.783 (0.758–0.805)	0.862 (0.818–0.904)	0.761 (0.730–0.787)	0.634 (0.589–0.674)
Saitama	0.879 (0.855–0.901)	0.737 (0.710–0.762)	0.879 (0.836–0.918)	0.699 (0.667–0.728)	0.585 (0.538–0.625)
Chiba	0.862 (0.831–0.891)	0.761 (0.733–0.787)	0.825 (0.774–0.874)	0.745 (0.713–0.773)	0.572 (0.520–0.617)

Data from the top six prefectures of Japan data (2013–2015) were analyzed.

*XGBoost* eXtreme gradient boosting, *AUROC* area under the receiver operating characteristic curve, *CI* confidence interval.

**Figure 2:**
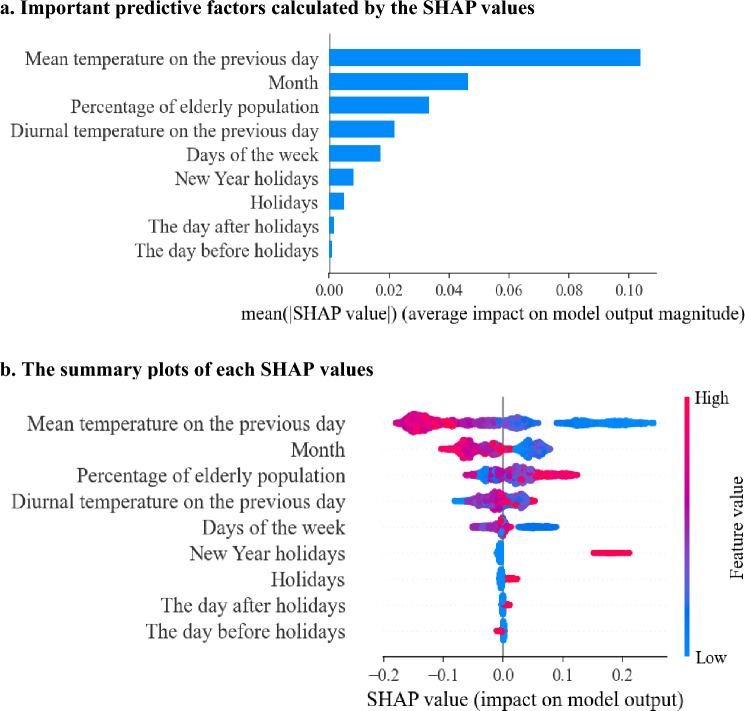
The SHAP value of OHCA prediction in the Tokyo training cohort. (**a**) Important predictive factors calculated by the SHAP values. (**b**) The summary plots of each SHAP values. A higher value of the feature value indicates a redder data point, and a lower value indicates a bluer data point. SHAP; SHapley Additive explanation, OHCA; out-of-hospital cardiac arrest.

In the analysis to search for important features using the SHapley Additive exPlanations (SHAP), the following five major features contributed significantly to the predictive model of OHCA: “mean temperature on the previous day,” “month,” “percentage of the elderly population,” “diurnal temperature on the previous day,” and “days of the week” (Fig. 2a). The violin plot of SHAP showed that a lower mean temperature on the previous day was associated with a higher risk of OHCA (Fig. 2b). Contrary to this, several redder points of “mean temperature” are on the positive SHAP value side; we observed that the higher mean temperature raised the possibility of OHCA high-incidence days and found that those data were in the summer season (June, July, August, and September). This reflects the non-linear correlation between the mean temperature and OHCA incidence. The SHAP values of the monthly variables (with November coded as the reference) show that the bluer points positively impact the model output. This implies that the winter season (December, January, and February) is an OHCA high-incidence time. The bluer on the right side of the “days of the week” variable results from the fact that OHCA occurrence on Sunday and Monday is frequent in our data.

### Comparison of OHCA incidence between elderly and non-elderly patients

Since the SHAP analysis revealed that “mean temperature on the previous day” and “percentage of the elderly population” were important factors, we divided the Tokyo data from 2005 to 2015 into elderly and non-elderly patients and plotted OHCA incidence per one million by temperature on the previous day (Supplementary Fig. 2a). In the elderly group, the non-linear (“U-shape”) correlation drastically changed with temperature changes, while the little effect of temperature on OHCA incidence was observed in the non-elderly group (Supplementary Fig. 2a, Supplementary results). According to the analysis of OHCA incidence on days of the week in each group, the OHCA incidence was high on Sundays and Mondays in the elderly group. At the same time, it was high only on Mondays in the non-elderly group (*p* < 0.01) (Supplementary Fig. 2b). There was no difference in the OHCA incidence in the months between the groups (Supplementary Fig. 3).

## Discussion

In this study, we developed a machine learning model to predict “risky” days for OHCA events using the meteorological and chronological data with a combination of nationwide OHCA repository data. Our algorithms were able to accurately predict high OHCA incidence days using XGBoost, which demonstrated that the incidence of OHCA was affected by weather conditions, the percentage of the elderly population, and calendar dates. In addition, the atmospheric temperature had a more substantial influence on OHCA events in the elderly than in the non-elderly population. Sunday and Monday were predicted to be the highest incidence days of OHCA for elderly people, while for the non-elderly, that was only Monday.

The benefit of using machine learning models in resuscitation was well established in literature: the deep learning model was developed to predict cardiac arrest and acute respiratory failure occurring in intensive care units more accurately^13^ than the National Early Warning Score (NEWS) and the Modified Early Warning Score (MEWS)^14^. Similarly, the machine learning model for predicting the daily OHCA incidence from meteorological and chronological data has also been proposed^15^. However, these studies used only a few algorithms for the accurate prediction of OHCA incidence and did not evaluate the high-incidence days of OHCA. Because the population and industry of each Japanese prefecture differ, and the Japanese climate has subpolar, temperate, and subtropical zones, there are some chronological changes and meteorological differences in each prefecture that need to be considered. We selected data from Japanese metropolitan areas, the weather conditions of which are not extremely different from Tokyo, and developed a prediction model based on the collected Tokyo data and verified its validity by applying it to the OHCA data of Tokyo and five other prefectures in Japan, which resulted in high accuracy of AUROC > 0.86. The XGBoost model also maintained high predictive accuracy for a higher occurrence of OHCA high-incidence days and high-incidence days in presumed patients of cardiac diseases. Therefore, our prediction algorithms are expected to aid in the identification of a high OHCA risk patient with various characteristics; including witness, not witness, and a variety of etiology. If an OHCA forecasting system based on machine learning algorithms could be established, it could alert local citizens, public utilities, and emergency services the day with a high risk of OHCA occurrences, and this could lead to strengthened monitoring of latent patients and prompt recognition and treatment indications for OHCA. This can be similar to hay fever and heat stroke forecasts.

Our SHAP analysis revealed that the major factors affecting OHCA incidence were low mean ambient temperature, the diurnal temperature of the previous day, the month, the percentage of the elderly population, and the day of the week. The result showing the link between low average temperature in the winter season and high OHCA incidence is in line with that reported in previous studies^16–18^. Even though exposure to cold weather was a major factor in the seasonality of OHCA incidence, the incidence peaked in January rather than February, in which the average temperature was the lowest of the year. It can be inferred that this may be related to seasonal changes in social activities and the new year holiday in January, which strongly influences OHCA occurrence, for example, holiday drinking^8^. A previous study showed that a large change in ambient temperature could be related to an increased sympathetic tone and blood viscosity, which may lead to an increased number of OHCA events^15, 19^. Changes in behaviour may explain circaseptan variability in OHCA incidence during weekdays and seasons^8^.

We focused on the elderly and non-elderly patients to clarify the influence of age on OHCA incidence. The comparison of the incidence of OHCA between the elderly and non-elderly groups demonstrated that the average temperature of the previous day more severely impacted OHCA incidence in the elderly than in the non-elderly group. This result is consistent with previous studies. Yoshinaga et al. reported that the number of cardiogenic OHCA cases in the elderly population increased after exposure to cold temperatures^17^.

The OHCA incidence was highest on Mondays for both the elderly and non-elderly populations, as well as on Sundays only for the elderly population. The reason for the increase in Mondays could be a change in the human behaviour pattern from weekends to weekdays, especially for workers, for example, “Mondays are depressing”^17, 20, 21^. Therefore, the reason for the high OHCA incidence on Sundays in the elderly group may be different from that in the non-working group.

Our study has several limitations. First, this was a nationwide OHCA study conducted in a single urban region of Japan. Therefore, whether the algorithm has a high predictive value in different areas with different backgrounds and meteorological data remains unclear. Second, detailed data on OHCA patients are unavailable, including the history of a medical condition, working, and behaviour patterns before OHCA. Third, some patients recognized more than half a day after the occurrence of cardiac arrest might be enrolled in this study because the judgements of transportation of the patients were dependent on the personnel of emergency medical personnel at the pre-hospital site. Finally, we excluded pediatric patients; hence, the model cannot be applied to children. Further studies including wider regions or detailed data on patient characteristics may strengthen the findings for the prediction of OHCA using machine learning.

In conclusion, we successfully predicted the days of the high incidence of OHCA from climate data using machine learning. Predicting the occurrence of OHCA may lead to the prevention or early detection of OHCA and therefore potentially improve its prognosis.

## Supplementary Information

Supplementary Information.

## Data Availability

The datasets used and/or analyzed during the current study are available from the corresponding author upon reasonable request.
