# Supplementary material for: Machine learning algorithms for predicting days of high incidence for out-of-hospital cardiac arrest
Source: Sci Rep. 2023 Jun 19;13:9950. doi: 10.1038/s41598-023-36270-6 (PMC10279733; doi:10.1038/s41598-023-36270-6)
Supplement: Supplementary file 1 — Supplementary Information. [file 41598_2023_36270_MOESM1_ESM.pdf]

Machine learning algorithms for predicting days of high incidence for out-of-hospital cardiac arrest

Kaoru Shimada-Sammori[1], MD; Tadanaga Shimada[1], MD, PhD; Rie E. Miura[1,2], PhD; Rui Kawaguchi[1], MD, PhD; Yasuo Yamao[1,2], Taku Oshima[1], MD, PhD; Takehiko Oami[1], MD, PhD; Keisuke Tomita[1], MD, PhD; Koichiro Shinozaki[1,3], MD, PhD; *Taka-aki Nakada[1,2], MD, PhD.

Affiliations:

1. Department of Emergency and Critical Care Medicine, Chiba University Graduate School of Medicine, Japan
2. Smart119 Inc, 2-5-1, Chuo, Chiba Japan
3. Department of Emergency Medicine, Zucker School of Medicine, New York, United States

Contact information of authors:

Kaoru Shimada-Sammori: kaorun.run.5@gmail.com
Tadanaga Shimada: tadanaga2000@gmail.com
Rie E. Miura: rmiura@smart119.biz
Rui Kawaguchi: ruka0704@icloud.com
Yasuo Yamao: yamao@cereja.co.jp
Taku Oshima: t_oshima@chiba-u.jp
Takehiko Oami: seveneleven711thanks39@msn.com
Keisuke Tomita: tomamu16@hotmail.co.jp
Koichiro Shinozaki: shino@gk9.so-net.ne.jp
*Taka-aki Nakada: taka.nakada@nifty.com

Correspondence: Taka-aki Nakada**.** Department of Emergency and Critical Care Medicine, Chiba University Graduate School of Medicine**,** 1-8-1 Inohana, Chuo, Chiba, 260-8677, Japan, Phone: +81-43-226-2372, Fax: +81-43-226-2371**,** Email: takanakada0@gmail.com

Supplementary Figure 1.

The location of six prefectures in this study in Japan

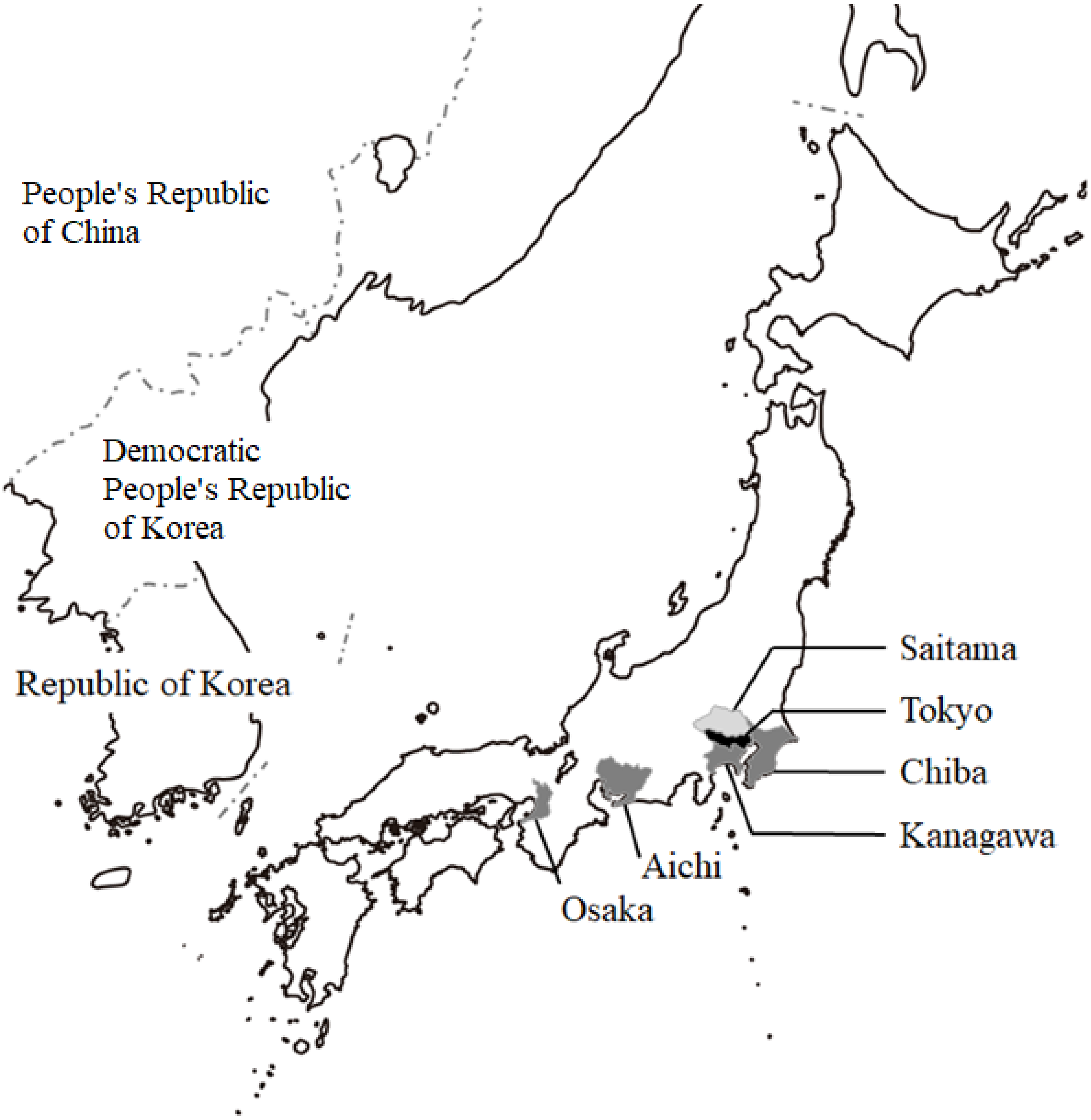

Tokyo; Latitude: 35 degrees north; Longitude: 139 degrees east

Saitama; Latitude: 34 degrees north; Longitude: 136 degrees east

Chiba; Latitude: 35 degrees north; Longitude: 140 degrees east

Kanagawa; Latitude: 35 degrees north; Longitude: 139 degrees east

Aichi; Latitude: 35 degrees north; Longitude: 136 degrees east

Osaka; Latitude: 34 degrees north; Longitude: 135 degrees east

Supplementary Table 1. Predicting the value of high-OHCA-incidence days presumed caused by cardiac diseases with the XGBoost model in the test data of the top-six population prefectures

	AUROC (95%CI)	Accuracy	Sensitivity	Specificity	F1-score
Tokyo	0.810 (0.782-0.837)	0.752 (0.724-0.778)	0.736 (0.695-0.775)	0.762 (0.727-0.797)	0.709 (0.676-0.741)
Kanagawa	0.817 (0.791-0.842)	0.736 (0.711-0.761)	0.784 (0.742-0.826)	0.713 (0.680-0.746)	0.656 (0.615-0.691)
Osaka	0.839 (0.810-0.864)	0.737 (0.710-0.762)	0.821 (0.778-0.860)	0.704 (0.672-0.733)	0.636 (0.596-0.674)
Aichi	0.859 (0.833-0.882)	0.730 (0.705-0.755)	0.849 (0.804-0.891)	0.687 (0.656-0.716)	0.625 (0.586-0.664)
Saitama	0.821 (0.797-0.848)	0.746 (0.721-0.771)	0.795 (0.757-0.831)	0.716 (0.682-0.749)	0.706 (0.675-0.735)
Chiba	0.790 (0.757-0.820)	0.710 (0.683-0.737)	0.773 (0.724-0.819)	0.685 (0.653-0.717)	0.596 (0.555-0.636)

Data from the top six prefectures of Japan data (2013-2015) were analyzed.
XGBoost, eXtreme Gradient boosting; AUROC, area under the receiver operating characteristic curve; CI, confidence interval

Supplementary Figure 2. OHCA incidence by temperature and day of the week in elderly and non-elderly patients.

a.

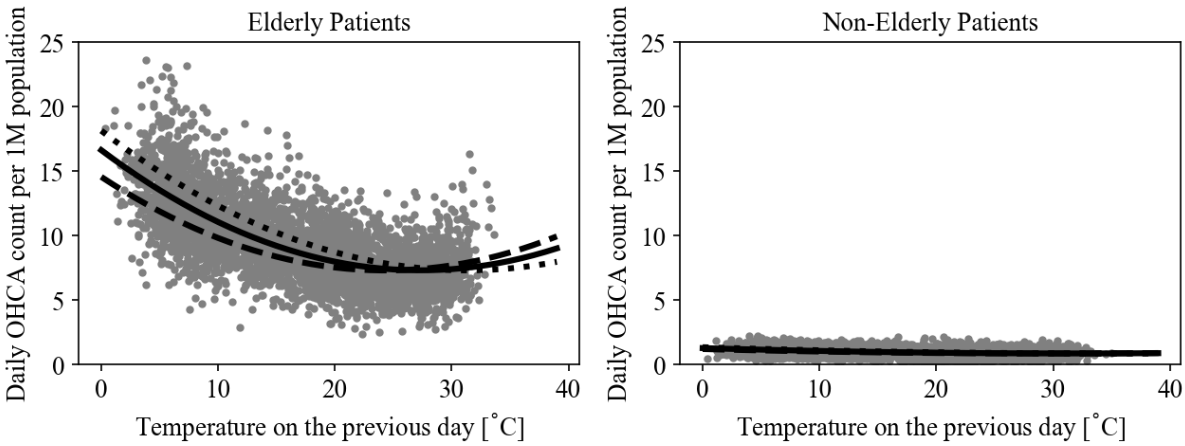

b

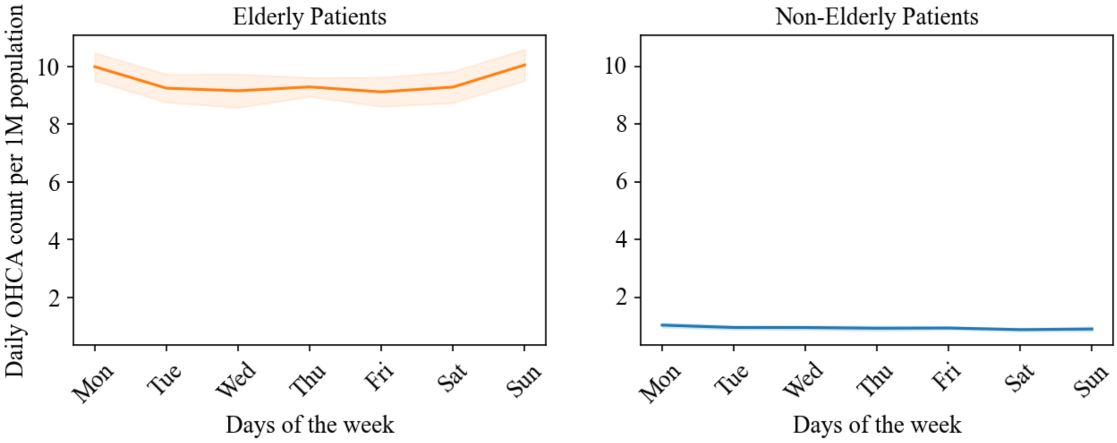

a. OHCA incidence by temperature in elderly and non-elderly patients.

The scatter diagrams show the relationships between the temperature on the previous day and OHCA incidence for the elderly patient groups (left) and the non-elderly patient groups (right). The y-axis of the left panel shows the daily OHCA count per 1 million elderly (or non-elderly) individuals. The solid, dashed, and dotted lines are fitted polynomial regressions (2nd order) with the mean, minimum, and maximum temperatures on the previous day, respectively.

b. OHCA incidence by day of the week in elderly and non-elderly patients.

Line charts show the mean OHCA incidence per day of the week. The shaded area represents the range between one standard deviation above the mean and one below the mean.

OHCA, out-of-hospital cardiac arrest; Mon, Monday; Tue, Tuesday; Wen, Wednesday; Thr, Thursday; Fri, Friday; Sat, Saturday; Sun, Sunday.

Supplementary Figure 3

OHCA incidence by month in elderly and non-elderly

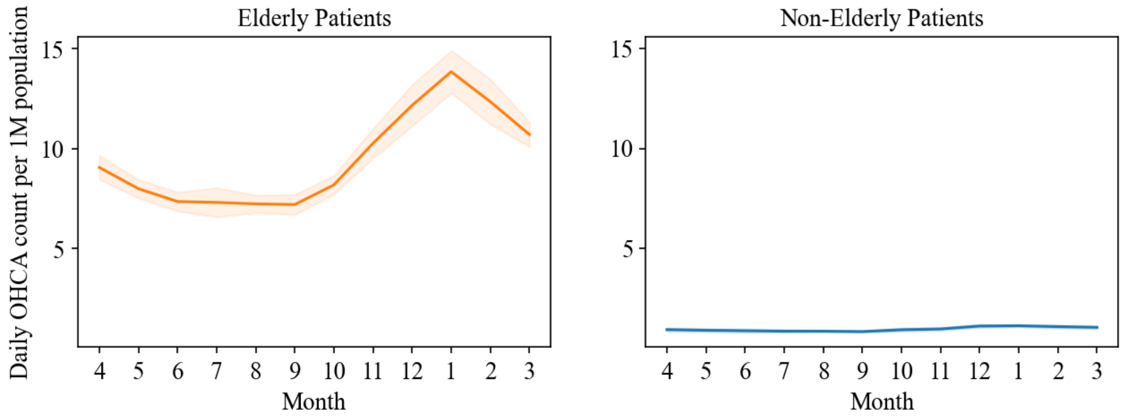

The incidence of daily OHCA was compared among months in the elderly and non-elderly patient groups. The line charts show the mean OHCA incidence per month for the elderly patient groups (left) and non-elderly patient groups (right). The vertical axis represents the daily OHCA count per 1 million elderly (or non-elderly) population. The shaded area represents the range between one standard deviation above the mean and one below the mean. There was no difference in the effect of the month on OHCA incidence between the two groups.

The elderly population was defined as people aged 65 and over.

OHCA; out-of-hospital cardiac arrest

Supplementary Methods

Feature Selection

1.) (***Transform non-linear to linear relation***) Several past studies have widely known the non-linear relation between meteorological variables and OHCA (reference). However, statistical analyses, such as the Granger causality test that we use in later steps, can fail in the presence of non-linearity. To transform the non-linearity correlation of meteorological variables with OHCA to linearity, we used univariate Poisson regression models of each meteorological variable to predict daily OHCA.

2.) (***Estimate Time-lag***) The meteorological factors would not affect the human body immediately but do with some time lag. After transforming the non-linear relationship to linear, we used the Granger causality (GC) test with the Walt test to filter out the meteorological variables whose prior values can predict the future values of OHCA. The impulse response function can be used to estimate the best lags of each variable impacting OHCA. We built impulse response functions with a maximum lag of 14 days using the OCHA and each variable. The best lags were determined when the response was significant at the 95% confidence level.

3.) (***Determine gradual effect using Exponential Weighted Mean***) We hypothesize that the changes in weather conditions during the past days gradually affect the human body. To capture this pattern, we used an exponential weighted mean (EWM) with the determined lagged values to give more recent observations more weights, while the older observations have less weights. The EWM function was computed on the time series shifted by the time-lag, l, and defined as: $\frac{x_{t-l}+(1-\alpha)x_{t-l-1}+(1-\alpha)^2x_{t-l-2}+(1-\alpha)^3x_{t-l-3}+\cdots}{1+(1-\alpha)+(1-\alpha)^2+\cdots}$, where x_t is a metrological variable at time t, α is a parameter which controls how the weighted mean decays. The best α was determined between 0.1 and 1.0 with a step of 0.1 from the

univariate Poisson model (using XGBoost), which minimized Poisson deviance. The mean and standard error were obtained using 10-fold cross-validation.

4.) (***Select the best predictor***) At this step, a variable with the largest impact on OHCA is selected by the minimum Poisson deviance, which is the mean ambient temperature. We updated the non-linearity relationship between the mean ambient temperature and daily OHCA to the one that took into account lag and α using a univariate Poisson model. Note that although the minimum and maximum temperatures can be selected owing to the minimum Poisson deviance, we chose the mean ambient temperature because of the slightly better mean Poisson deviance than the others. They are highly correlated with the mean temperature; thus, we did not consider them candidates in further steps. The existing strong collinearity in a model is not only redundant but also might cause the worse performance of the model and thus should be removed in advance.

5.) (***Repeat 1-4***) To minimize the impact of mean temperature on OHCA, we considered the causality of any other variables to the residual, which was obtained by subtracting the predicted values of OHCA using the univariate regression model from the observed values. We then repeated the above steps until the Poisson deviance did not improve. Finally, we selected the best combination of two variables: the mean average temperature with a lag of 1 day and $\alpha = 0.4$ and the diurnal temperature with a lag of 1 d and $\alpha = 1.0$

Data Engineering

The month and days of week variables converted to the cyclic variables

When the models were trained, the month and days of the week variables were transformed into their corresponding sine and cosine functions to express their periodic nature. Each variable is defined as follows: month (cosine) = cos(month/12 * π * 2), month (sines) =

sin(month/12 * π * 2), weekday (cosine) = cos(weekday/7 * π * 2), and weekday (sines) = sin(weekday/7 * π * 2).

Supplementary results

The mutual Information value of OHCA incidence by the temperature of the week in elderly and non-elderly patients.

We determined the mutual information (MI) value for the elderly and non-elderly groups to assess the strength of the non-linear relationship between temperature change and OHCA incidence; the MI value was 0.318 for the elderly group and 0.07 for the non-elderly group, which was lower than that of the elderly group. Thus, the binary association was stronger in the older group.
